# Presence of Foodborne Bacteria in Wild Boar and Wild Boar Meat—A Literature Survey for the Period 2012–2022

**DOI:** 10.3390/foods12081689

**Published:** 2023-04-18

**Authors:** Caterina Altissimi, Clara Noé-Nordberg, David Ranucci, Peter Paulsen

**Affiliations:** 1 Department of Veterinary Medicine, University of Perugia, Via San Costanzo 4, 06121 Perugia, Italy; caterina.altissimi@studenti.unipg.it (C.A.); david.ranucci@unipg.it (D.R.); 2 Esterhazy Betriebe GmbH, Esterházyplatz 5, 7000 Eisenstadt, Austria; c.noe-nordberg@esterhazy.at; 3 Unit of Food Hygiene and Technology, Institute of Food Safety, Food Technology and Veterinary Public Health, University of Veterinary Medicine Vienna, Veterinärplatz 1, 1210 Vienna, Austria

**Keywords:** wildlife, game meat, *Salmonella*, *Listeria*, *Campylobacter*, *Yersinia*, mycobacteria, verotoxinogenic *E. coli*, *Brucella*, *Staphylococcus aureus*

## Abstract

The wild boar is an abundant game species with high reproduction rates. The management of the wild boar population by hunting contributes to the meat supply and can help to avoid a spillover of transmissible animal diseases to domestic pigs, thus compromising food security. By the same token, wild boar can carry foodborne zoonotic pathogens, impacting food safety. We reviewed literature from 2012–2022 on biological hazards, which are considered in European Union legislation and in international standards on animal health. We identified 15 viral, 10 bacterial, and 5 parasitic agents and selected those nine bacteria that are zoonotic and can be transmitted to humans via food. The prevalence of *Campylobacter*, *Listeria monocytogenes*, *Salmonella*, Shiga toxin-producing *E. coli*, and *Yersinia enterocolitica* on muscle surfaces or in muscle tissues of wild boar varied from 0 to ca. 70%. One experimental study reported the transmission and survival of *Mycobacterium* on wild boar meat. *Brucella*, *Coxiella burnetii*, *Listeria monocytogenes*, and Mycobacteria have been isolated from the liver and spleen. For *Brucella*, studies stressed the occupational exposure risk, but no indication of meat-borne transmission was evident. Furthermore, the transmission of *C. burnetii* is most likely via vectors (i.e., ticks). In the absence of more detailed data for the European Union, it is advisable to focus on the efficacy of current game meat inspection and food safety management systems.

## 1. Introduction

During the last decade, numbers of wild ungulates, in particular wild boars, have been rising significantly worldwide, generating environmental, economic, public health, and social concerns. Wild boar is the most widespread species due to its high adaptability and fertility rate, and its spread has been facilitated by climate change, the abandonment of rural areas, reforestation, a lack of predators, animal introductions, and supplementary feeding for hunting purposes [1,2,3,4]. The high density of this expanding species is causing, in particular, in Europe, not only relevant damages to agriculture and ecosystems and an increase in road accidents but also increases the risk of transmission of pathogens from wild boar to humans, livestock, and domestic animals [5,6]. The synanthropic behavior of wild boars is an important co-factor in creating disease-transmission scenarios [7]. Furthermore, the attention being paid to wild boar population control is leading to an increase in the availability of game meat. Additionally, the market has to face different harvesting practices, the wider distribution of this product, and, simultaneously, guarantee its safety aspects. In this context, it is of the utmost importance to understand the epidemiological situation and the major hazards due to the consumption of such meat.

Indeed, it has been highlighted by several authors how wild boar could act as a reservoir, playing an important role in the maintenance, circulation, and diffusion of certain pathogens for humans and animals [8,9,10,11,12]. In particular, the same authors focused their attention on the most relevant bacterial food hazards that: cause disease to wild boar and can be present in the meat (e.g., *Brucella* spp., *Mycobacterium tuberculosis* complex); are harbored in the gut or other tissues and then transferred to the meat during processing (e.g., *Salmonella* spp., *Campylobacter* spp., *Escherichia coli*, *Yersinia enterocolitica*); contaminate the carcass due to their presence on animal skin and in the environment (e.g., Listeria spp., *Staphylococcus aureus*).

In a framework of global health, it is essential to consider not only zoonotic diseases but also animal diseases with an impact on food security. The aim of this review is to give an overview of publications from the period 2012–2022 on the presence of biological hazards in the wild boar population. In particular, foodborne zoonotic bacteria commonly reported in meat from domestic animals will be the focus, and their presence in wild boars will be reviewed.

## 2. Materials and Methods

A list of infectious agents was compiled, combining zoonotic agents included in compulsory monitoring in the European Union (Directive 2003/99/EC List A) [13], zoonotic agents monitored according to the epidemiological situation (Directive 2003/99/EC List B) [13], swine and multiple species diseases, infections, and infestations listed by the World Organisation for Animal Health (OIE), and the most common agents responsible for foodborne outbreaks reported from the European Food Safety Authority (EFSA) during the period 2015–2020 and in the EU Rapid Alert System for Food and Feed (RASFF).

For each agent on the list, a literature search was conducted on SCOPUS using the name of the selected pathogen or the related disease combined with the search string: “wild” AND “boar” OR “feral AND pig” OR “warthog”. During the literature search, biological hazards that do not concern wild boars were excluded. The search was then adjusted for (i) the time period 2012–2022, (ii) document type as article or review, and (iii) English as the selected language. Papers about the prevalence and control strategy of selected diseases were considered, whereas articles reporting solely detection methods were included only if relevant for the interpretation of results. Although our work focuses on the relevance of wild boar (meat) in the European Union, we included references from other countries in view of imports of wild boar meat from third countries in the EU; similarly, studies on feral pigs and warthogs were included.

We also report the number of publications per agent and year as a proxy for the relevance of the agent and the interest and effort of the scientific community in this topic [14]. From this long list of biological hazards specifically addressed in national legislation or by international organizations, we selected those with evidence that they are actually transmitted via the handling, processing, and consumption of porcine meat and meat products.

## 3. Results

### 3.1. Overview of Biological Hazards in Wild Boar and Their Impact on Food Safety and Security

The array of biological agents addressed in EU legislation and international organizations such as the OIE is displayed in Table 1. Information on zoonotic potential and mode of transmission was taken from OIE, EFSA, and ECDC documentation. Notably, not all agents are zoonotic, and not all zoonotic agents are transmitted by meat. Among the pre-selected (i.e., taken from EU and OIE documents) infectious agents, no scientific literature was retrieved for two viruses and one bacterial genus. A clear increase (i.e., more than one doubling) in the average number of publications per year in the period 2017–2022 compared with that from 2012–2017 was noted for the viral diseases African swine fever, West Nile fever, and Japan encephalitis; the bacterium *Listeria*; and the parasite genera *Cryptosporidium*, *Cysticercus*, and *Echinococcus*.

For a detailed review of the occurrence and significance of biological hazards, we focused on bacteria since these are the main causative agents for foodborne diseases reported in the EU [15].

### 3.2. Occurrence and Prevalence of Selected Zoonotic Bacteria in Wild Boar

#### 3.2.1. *Brucella*

Brucella (B.) are gram-negative, nonsporeforming, aerobic, short-rod bacteria that include several pathogenic species. In the EU, monitoring of brucellosis is compulsory (Directive 2003/99/EC List A) [13]. In ruminants, swine, and dogs, infection with the agents causes diseases of the reproductive system, e.g., abortion or epididymitis. Symptomless carriers can excrete the pathogen, e.g., via milk. Small ruminants with mastitis caused by Brucella-melitensis can excrete the pathogen via milk. Ingestion of raw milk, inhalation, or close contact with infected animals or parts thereof (e.g., when dressing hunted wild game) can lead to human infections. These may resemble a feverish flu, whereas more severe courses involve splenomegaly and splenic or hepatic abscesses. In 2021, cattle livestock in 21 EU member states was officially free from brucellosis (*B. abortus*, *B. melitensis*, and *B. suis*), and as regards small ruminant livestock, 20 member states were officially free from the pathogen. In 2021, 162 human cases were reported, two of them foodborne. In 2020, there were also 2 cases linked to the consumption of sheep meat products, with *B. melitensis* being the causative species [15]. In the EU rapid alert system for food and feed (RASFF), no notification of the presence of Brucella in food was found.

As regards wild boar and Brucella, 96 documents were retrieved. Those reporting prevalence data were included in Table 2 (seropositivity) and Table 3 (DNA or viable bacteria). With respect to serological testing, the cross-reactivity with the Yersinia enterocolitica O9 antigen is a well-known issue. More recent methods may overcome this problem [16]. Some authors present seroprevalences corrected for cross-reactivity [17]. When tissues/organs of the animal were tested by bacteriological culturing, or PCR, blood, lymphatic organs, genital organs, and fetuses were examined. There was no study on Brucella in muscle tissue or commonly consumed organs, e.g., liver, from wild boar. When Brucella species and biovars are explicitly reported, it is mainly B. suis biovar 2.

While no documented cases of meat-borne brucellosis could be retrieved, several cases of brucellosis in humans hunting wild boar and dressing wild boar carcasses have been published; most reports are from the USA [18,19,20,21], but also from France [22] and Australia [23]. In two cases, neurological disorders [18,23] were reported, and in one case, arterial and venous thromboses were reported [20], which are otherwise rarely observed [24]. Similarly, dogs frequently in contact with wild boar are at risk of seropositivity to Brucella [25,26,27].

**Table 2:** Prevalence of *Brucella* spp. antibodies in wild boars (2012–2022), by country and continent.

Prevalence/Frequency	Species	Matrix	Country	Comment	Ref.
15.6% (15/96)	*B.* spp.	Sera	Italy (Tuscany)	serology	[28]
5.74% (16/287)	*B*. spp.	Sera	Italy (Tuscany)	RBT, CFT	[29]
5.1% (22/434) 13.5% (58/434)	*B.* spp.	Sera	Italy (Campania)	RBT ELISA	[30]
0.53% (2/374)	*B.* spp.	Sera	Italy (Tuscany)	RBT, CFT	[31]
6.2% (35/570)	*B.* spp.	Sera	Italy (Sardinina)	ELISA	[32]
15% (19/126)	*B. suis*	Sera	Italy (Central)	serology	[33]
59.3% (121/204)	*B.* spp.	Sera	Spain (Extremadura)	ELISA	[34]
9.4% (45/480)	*B. suis* biovar 2	Sera	Serbia	RBT, ELISA	[35]
1.3% (42/3230)	*B*. spp.	Sera	Croatia	RBT; CFT; ELISA	[36]
6.4% (131/2057)	*B.* spp.	Sera	Netherlands	ELISA	[37]
0% (0/286)	*B. suis*	Blood	Sweden	ELISA	[38]
9% (8/87)	*B*. spp.	Blood	Finland	RBT, ELISA; visceral organs from 5 seropos. animals available, in 4 of which *B. suis* biovar 2 was detected	[39]
13.3% (139/1044)	*B. suis*	Sera	Latvia	RBT, CFT, ELISA, data corrected for O9-cross-reactivity	[17]
0% (0/100)	*B*. spp.	Sera	South Africa	Warthog	[40]
12.5% (1/8)	*B*. spp.	Sera	Kenya	Warthog; Antibody-ELISA	[41]
0% (0/86)	*B*. spp.	Sera	Brazil	Agglutination, 2MET	[42]
0% (0/61)	*B*. spp.	Sera	Brazil (Santa Catarina)		[43]
0.49% (1/205)	*B*. spp.	Blood	Brazil	Feral pigs; serology (BAPA, FPT)	[44]
0% (0/15)	*B*. spp.	Blood	Colombia	Feral pigs	[45]
2.2% (1/46)	*B*. spp.	Blood	Guam	Feral pigs; FPT	[46]
0.7% (2/282)	*B. abortus*	Sera	USA (Oklahoma)	BAPA, RIV, FPT	[47]
2.95% (7/238)	*B. suis*	Sera	Australia (NSW)	RBT, CFT	[48]
9.6% (8/83)	*B* *. suis*	Blood	Australia (Queensland)	RBT, CFT	[49]
0% (0/303)	*B*. spp.	Sera	Finland	RBT	[50]
54.9% (641/1168)	*B.* spp.	Sera	Belgium	ELISA	[51]

BAPA = Buffered Acidified Plate Antigen, CFT = Complement Fixation Test, RBT = Rose-Bengal-Test, RIV = Rivanol Agglutination, 2MET = 2-Mercapto-Ethanol.

**Table 3:** Prevalence of *Brucella* spp. (viable bacteria or DNA) in wild boar (2012–2022), by country and continent.

Prevalence/Frequency	Species	Matrix	Country	Comment	Ref.
12.5% (1/8)	*B*. spp.	Sera	Kenya	Warthog; PCR	[41]
1.4% (4/287) 1.7% (5/287) 2.2% 0% (0/287)	*B. suis* biovar 2	Lymph nodes epididymides fetuses pooled livers, spleens	Italy (Tuscany)	DNA	[29]
0.83% (2/240)	*B*. spp.	Inner organs	Denmark	culture	[52]
3.8% (7/180) 10.5% (19/180)	*B.* spp.	Tonsils	Netherlands	culture PCR; confirmed as *B. suis* biovar 2	[37]
22% (19/87)	*B. suis*	Feces	USA (Georgia)	Feral pigs, PCR	[53]
1.3% (5/389)	*B. suis* biovar 2	Retropharyngeal lymph nodes	Italy	culture	[54]
3.7% (7/188)	*B. suis* biovar 2	Reproductive organs	Spain (Extremadura)	culture, PCR	[34]
0% (0/238)	*B*. spp.	Blood	Australia (NSW)	culture	[48]

#### 3.2.2. *Campylobacter*

*Campylobacter* is a genus of gram-negative, nonsporeforming, microaerophilic, motile spiral-shaped bacteria, with *C. jejuni* and *C. coli* as the main species involved in Campylobacteriosis. The principal symptoms of *Campylobacter* infections are diarrhea, abdominal pain, fever, headache, nausea, and vomiting. The disease is usually self-limiting, and death is rare except in severe cases in elderly people, very young children, or immunocompromised patients [55]. In 2021, campylobacteriosis was the zoonosis with the highest number of human cases reported in the EU, with 127,840 cases of illness and 10,469 hospitalizations. With respect to foodborne outbreaks, it was the fourth most frequently reported agent with 249 outbreaks, 1051 cases, and 134 hospitalizations [15]. *Campylobacter* is common in food animals such as poultry, pigs, and cattle, and the main transmission route is via meat and meat products, as well as raw milk and milk products.

Twenty-two articles have been published from 2012 to 2022 regarding the prevalence of *Campylobacter* in wild boars, five of which were excluded as not relevant. The main matrix considered for the isolation of *Campylobacter* is feces, as reported in Table 4. The references highlighted the role of wild boars as a possible source of *Campylobacter* infection due to the prevalence of *Campylobacter* spp. in feces samples, albeit in a variable range from 12.5% [56] to 66% [57]. Several species have been isolated from fecal samples in varying prevalence ranges, e.g., *C. lanienae* from 1.2% [56] to 69% [58], *C. hyointestinalis* from 0.8% [59] to 22.1% [60], *C. coli* from 0.8% [56] to 16.3% [58], and *C. jejuni* from 0% [61] to 4.1% [58] of samples. As suggested by [59], the degree of urbanization of some areas populated by wild boars could have a relationship with the detection frequency of some *Campylobacter* species; in particular, *C. lanienae* was more frequently isolated in low urbanizations areas, suggesting that this pathogen could be interconnected with the kind of diet available.

During the period considered, only two studies were conducted on carcasses, and they presented similar results, with a prevalence of *Campylobacter* spp. of 11.1% [62] and 16.7% [63]. Peruzy et al. [64] investigated the presence of *Campylobacter* in wild boar meat samples, but the pathogen was not detected.

To date, the EU has set food processing hygiene criteria for *Campylobacter* only for poultry [65].

**Table 4:** Prevalence of *Campylobacter* spp. in wild boar (2012–2022) feces or on carcasses or meat.

Prevalence/Frequency	Species	Matrix	Country	Comment	Ref.
51.8% (29/56)	*Campylobacter* spp.	Feces	Italy		[63]
50% (38/76) 40.8% (31/76)	*Campylobacter* spp. *C. lanienae*	Feces	Italy	Campylobacter spp. with levels up to 10³ CFU/g was detected in 39.5% animals	[66]
66% (188/287)	*Campylobacter* spp.	Feces	Spain	One isolate was identified as *C. jejuni*	[57]
60.8% (79/130) 46.2% (60/130) 16.9% (22/130) 0.8% (1/130) 0% (0/130)	*Campylobacter* spp. *C. lanienae* *C. coli* *C. hyointestinalis* *C. jejuni*	Feces	Spain	4% WB had both *C. lanienae* and *C. coli*, and 1% had both *C. lanienae* and *C. hyointestinalis*. All the isolates were resistant to at least one antimicrobial agent considered	[59]
38.9% (49/126) 69.4% (34/49) 16.3% (8/49) 4.1% (2/49)	*Campylobacter* spp. *C. lanienae* *C. coli* *C. jejuni*	Feces	Spain		[58]
19.51% (8/41) 4.88% (2/41) 0% (0/41)	*Campylobacter* spp. *C. coli* *C. jejuni*	Feces	Spain		[61]
43.8% (53/121) 25.6% (31/121) 17.4% (21/121) 0.8% (1/121)	*Campylobacter* spp. *C. lanienae* *C. hyointestinalis* *C. jejuni*	Feces	Japan	Five (16%) and 6 (29%) isolates of *C. lanienae* and *C. hyointestinalis*, respectively, were resistant to enrofloxacin	[67]
22.1% (71/321)	*C. hyointestinalis*	Feces	Japan		[60]
12.5% (31/248) 9.7% (25/248) 1.2% (3/248) 0.8% (2/248)	*Campylobacter* spp. *C. hyointestinalis* *C. lanienae* *C. coli*	Feces	Japan		[56]
3.5% (13/370) 1.6% (6/370)	*C. coli* *C. jejuni*	Feces	USA	*C. coli* was significantly more frequent in female feral pigs	[68]
0% (0/87)	*C. jejuni*	Feces	USA		[53]
16.7% (5/30)	*Campylobacter* spp.	Carcass	Italy		[63]
11.1% (4/36)	*Campylobacter* spp.	Carcass	Italy		[62]
0% (0/28)	*Campylobacter* spp.	Meat	Italy		[64]

WB = wild boars.

#### 3.2.3. *Coxiella burnetii*—Q-Fever

*Coxiella burnetii* is a gram-positive short-rod bacterium that grows aerobically within but also outside of host cells. It can form spores and persist under dry and acidic conditions. The bacterium is not only excreted via effluents, but several tick species can act as vectors for the pathogen. Infection of humans can occur via contact with effluents, ingestion of contaminated food, and inhalation of aerosolized pathogens, but also by tick bites. Infection causes a feverish disease (Q-fever) with pneumonia, followed by affections of the heart, liver, and spleen. In the EU, human cases are notifiable. Data indicate that the number of human cases as well as prevalence in animals is declining. However, monitoring of farm and wild animals is not harmonized in the EU [15]. At least 347 of the 460 confirmed human cases of Q-fever in 2021 were acquired within the EU, and the pathogen was prevalent in 5.2%, 5.9%, and 16.5% of samples from cattle, goats, and sheep, respectively. Since not all member states submitted data, the reported percentages are not necessarily representative of the EU [15]. Studies conducted on *C. burnetii* and wild boar can be grouped into three categories: (i) those on ticks collected from wild boars or from hunters or dogs in frequent contact with wild boars; (ii) those on serum or spleen samples from wild boar; and (iii) studies on the genetic diversity of *C. burnetii*.

Within Europe, studies originated in Spain and Italy (Table 5). DNA from *C. burnetii* was detected in 1.9% of spleen samples [69], and antibodies were found in 5.5% of serum samples [70] from wild boar in Spain. In studies from Italy, the pathogen was not recovered from wild boar samples but from ticks feeding on wild boars (0.5%; [71]) and from dogs in contact with wild boars (5.1%; [72]). Wild boar is not a specific or primary host for the pathogen [73], but since the agent is occasionally detected in tissues from wild boar, hunters and consumers handling and processing wild boar (meat) are both occupationally and dietary exposed. Similarly, hunters and dogs often in contact with wild boars are at risk of exposure to tick-borne pathogens, among them *C. burnetii* [71].

No notifications regarding the presence of *C. burnetii* in foods were listed in the EU rapid alarm system (RASFF).

#### 3.2.4. *Listeria monocytogenes*

Listeriosis is a zoonotic disease caused by *Listeria monocytogenes*, a gram-positive, nonsporeforming, facultatively anaerobic bacterium. Foodborne listeriosis is one of the most severe diseases, causing septicemia, neurologic disorders, and reproductive disorders. Pregnant women, elderly people, and individuals with weakened immune systems are at risk for severe courses of the disease. *Listeria* is a ubiquitous microorganism that thrives in soil, water, vegetables, and the digestive tracts of animals. It can survive and proliferate in different environmental conditions since it is tolerating a wide range of pH and temperatures [80]. The main transmission route of *Listeria* is through the ingestion of contaminated food [15].

Twelve studies have been found from 2012 to 2022 regarding the presence of *Listeria* spp. in wild boar carcasses, meat, and related products, two of which were excluded as not relevant (Table 6). *Listeria monocytogenes* was detected by many authors in tonsil samples, highlighting this organ as the preferred matrix for the presence and detection of *Listeria* [63,81,82]. Fredriksson-Ahomaa et al. [39] found *L. monocytogenes* in 48% of spleen and kidney samples from wild boars. Almost all isolates belonged to serotype 2a, except for two isolates identified as serotype 4b. The presence of *Listeria* in tonsils and in visceral organs underlines the necessity of particular attention during handling and evisceration of wild boar carcasses.

Regarding the presence of *Listeria* in wild boar meat products, Roila et al. [83] did not detect the pathogen in wild boar salami, whereas Lucchini et al. [84] isolated *Listeria* spp. in 65% of cured game meat sausages. Three species were identified: *L. monocytogenes,* 24%*; L. innocua*, 32% and *L. welshimeri,* 8%. Counts of *L. monocytogenes* were, however, always below the legal limit of 100 cfu/g set by Regulation (EC) 2073/2005 [65].

In the years 2020–2022, 340 notifications regarding the presence of *L. monocytogenes* in foods were listed in the EU rapid alarm system RASFF, of which 82 implicated meat and meat products; there was no explicit mention of game meat or wild boar meat in particular.

**Table 6:** Presence of *Listeria* sp. in wild boar, 2012–2022.

Prevalence/Frequency	Species	Matrix	Country	Comment	Ref.
0. 35% (1/287)	*L. monocytogenes*	Rectal swabs	Italy	*L.m.* serogroup IVb, serovar 4b; resistant to cefoxitin, cefotaxime and nalidixic acid	[85]
68.5% (37/54) 35.3% (18/51) 26.7% (8/30) 0% (0/30)	*Listeria* spp. *L. monocytogenes* *Listeria* spp. *L. monocytogenes*	tonsils tonsils Carcass Carcass	Italy	prevalence influenced by animal age and environmental temperature	[63]
48% (63/130)	*L. monocytogenes*	Spleen and kidneys	Finland		[39]
24.5% (12/49)	*L. monocytogenes*	Liver or tonsils or feces or intestinal lymph nodes, caecum content	Germany	Positive in at least one of the different matrices studied	[81]
14.3% (7/49)	*L. monocytogenes*	Tonsils	Germany		[81]
2% (1/49)	*L. monocytogenes*	Liver and intestinal lymph nodes and caecum content and feces	Germany	The same animal resulted positive for *L.m*. in all the matrices analyzed	[81]
51.8% (14/27) 40.7% (11/27) 0% (0/27)	*Listeria* spp. *L. monocytogenes* *L. monocytogenes*	Tonsils Tonsils Feces	Spain		[82]
37.3% (28/75) 0% (0/75)	*Listeria* spp. *L. monocytogenes*	Feces	Japan		[67]
0% (0/72)	*L. monocytogenes*	Carcass	Italy		[86]
65% (24/37) 24% (9/37) 32% (12/37) 8% (3/37)	*Listeria* spp. *L. monocytogenes* *L. innocua* *L. welshimeri*	Game meat cured sausages	Italy	*L.m.* < 10 cfu/g	[84]
0% (0/40)	*L. monocytogenes*	Wild boar salami	Italy		[83]

#### 3.2.5. *Mycobacterium tuberculosis* Complex

*Mycobacterium tuberculosis* complex is a group of *mycobacteria* that include *M. tuberculosis,* the major cause of human tuberculosis (TB), and other genetically related species that affect livestock and wild animals but are also implicated in human disease [87,88]. Among these species, in the last decade, *M. bovis* [89,90,91,92,93,94,95,96,97,98,99,100,101,102,103,104,105,106,107,108,109,110,111,112,113,114,115], *M. caprae* [89,104,111,116,117], and *M. microti* [118,119,120,121,122,123,124] have been frequently reported from wild boar, feral pigs, and warthogs in different countries.

The MTC bacteria can cause localized granulomas (primary complex) after entering the host through the respiratory or digestive tract, and when the organism´s immune system cannot contain it (which can be the case in the elderly, children, and in people with compromised immune systems), it may be followed by primary or secondary-reactivated TB. Meningitis, extrapulmonary granulomas, miliary tuberculosis, and other disseminated/generalized forms are only a few examples of the various manifestations, along with a variety of clinical symptoms [125]. *M. bovis* is usually transmitted through oral ingestion, and therefore the extrapulmonary lesions in humans are more frequent than for *M. tuberculosis* [126]. In wild boar, the main primary complex is usually located in the submandibular and retropharyngeal lymph nodes, where the MTC is most frequently isolated [89,98,105,117,122,127,128]. Lesions were also reported in the tonsils, lung, mediastinal lymph nodes, spleen, liver, and kidney [106,117,127,128]. The lesion in the lymph nodes is characterized by caseous or necrotic-calcified tubercles that are defined as tuberculosis-like lesions (TBLL), as other *mycobacteria* different from MTC (e.g., *M. avium subsp. hominissuis*) could cause the same lesion [119,129,130,131]. *M. bovis* and *M. caprae* could also be detected (isolated/PCR) in lymph nodes without visible lesions [94,105,128,131]. Wild boar is reported for MTC shedding through the oral, nasal, and fecal routes [132], and therefore animal aggregation areas could result in contaminated water and soil and the maintenance of the infection in wildlife and livestock [118,133,134].

In addition, 214 studies regarding MTC and non-MTC in wild *Suidae* species have been found in the literature over the considered period, but only 35 were related to prevalence studies of MTC and were therefore considered. These studies were performed both by serology (Table 7) and by isolation or direct identification of *mycobacteria* in organs and tissues (Table 8). The prevalence of MTC varies between countries and between regions/counties inside each nation (e.g., Spain), but also due to the investigated matrix and the diagnostic methods adopted [94,98,135]. In this context, some studies were performed to define the sensitivity of different diagnostic tools on sera and on organs and tissues [94,96,119,136]. The serological prevalence of MTC in wild boar is generally conducted over multi-year studies and ranged from 87.7% in Montes de Toledo and Doñana National Park (Spain) [132] to near 0% in the USA [137]. The prevalence of MTC isolation in tissue and organs, considering studies conducted on more than 100 subjects, ranges from 64.2% for *M. microti* in the Lombardia region (Italy) [123] to 1.1% for *M. bovis* in the Basque Country (Spain) [89].

The presence of MTC in wild boar is still recognized as one of the main barriers to the eradication of the disease in livestock and, subsequently, in humans, particularly when extensive pastoral systems are implemented and there is an interface between farmed and wild animals [93,100,101,104,111,133,138,139]. Although the disease is notifiable in many countries (such as Europe and the United States), its control in wild boar is primarily restricted to standard visual game meat inspection, which is thought to be insufficient to find primary complex and small lesions [117], especially as post-mortem inspection could be carried out also by trained hunters [EC Regulation 853/2004 [140]]. Even the cultural method for bacterial isolation is less effective than other diagnostic tools (e.g., screening PCR directly performed on target tissues, such as head lymph nodes, even when no TBLL are detected) [94,136]. Another topic to be considered is the free movement of wildlife that could spread the disease in different geographic areas. The identification and long-term monitoring of the genotype/spoligotype existing in a territory may aid in specific surveillance plans and control actions [100,141].

Despite the role of wild boar as a reservoir for MTC and the possible transmission through food [11], wild boar meat and meat products as a source for human infection are reported only by Clausi et al. [142]. In this study, PCR tests revealed the presence of MTC DNA on the carcass surface of wild boar without TBLL, but no *Mycobacterium* spp. could be isolated. Clausi et al. [142] added lymph nodes with active TBLL (*M. bovis*) to meat batter during sausage processing. Although live bacteria could be isolated only at day 23 after the contamination of the sausages (neither before nor after), bacterial DNA was detected (PCR) throughout the entire study period (end of sampling at day 41). When *M. bovis* (10^5^ CFU/g) was directly added during sausage manufacturing, it was isolated for up to 22 days of ripening. When meat surfaces were experimentally contaminated with *M. bovis,* the bacterium could be recovered after frozen storage for over 5 months [142]. The role of wild boar meat and derived raw meat products could therefore be further investigated, even if other authors consider meat a negligible source of human infection [117].

**Table 7:** Seroprevalence of MTC in wild boar, feral pigs, and warthogs, 2012–2022.

Prevalence/Frequency	Species	Country	Area	Comment	Ref.
16.7% (5/30)	*MTC*	Malaysia	Selangor	Sampling in 2019–2020 Test used: bovine purified protein derivative (bPPD)-based indirect in-house ELISA	[127]
17% (326/1902)	*MTC*	Spain	Basque Country	Sampling in 2010–2016 Test used: in house validated enzyme-linked immunosorbent assay (ELISA)	[143]
10.6% (46/434)	*MTC*	Italy	Campania Region	Sampling in 2012–2017 Test Used: Indirect ELISA INgezim Tuberculosis DR kit based on recombinant M. bovis protein (MPB83)	[92]
2.4% (16/278)	*MTC*	Portugal	Several Counties	Sampling in 2006–2013 Test used: bPPD-based indirect in-house ELISA	[95]
49.0% (49/100)	*M. bovis*	South Africa	uMhkuze Nature Reserve in Kwa-Zulu Natal, Marloth Park on the southern border of Kruger National Park in Mpumalanga	Sampling in 2013–2015 Test used: Indirect PPD ELISA and TB ELISA-VK^®^	[96]
87.7% (36/41)	*MTC*	Spain	Montes de Toledo and Doñana National Park	Sampling in 2011–2013 Test used: bPPD-based indirect in-house ELISA Prevalence was obtained adding the number of animals with lesions at necroscopy to the number of positive serological samples	[132]
0.0003% (1/2735)	*MTC*	USA	National survey	Sampling in 2007–2015 Test used: bPPD-based indirect ELISA	[137]
2.4% (18/743)	*MTC*	Switzerland	Geneva, Mittelland, Jura, Thurgau, Tessin	Sampling in 2008–2013 Test used: bPPD-based indirect in-house ELISA	[109]
5.9% (123/2080)	*MTC*	France	58 Departments	Sampling in 2000–2004/2009–2010 Test used: bPPD-based indirect ELISA	[144]
2.1% (22/1057)	*MTC*	Spain	Asturias and Galicia	Sampling in 2010–2012 Test used: bPPD-based indirect ELISA	[111]
67.7% (87/130)	*MTC*	Spain	Andalusia	Sampling in 2006–2010 Test used: MPB83-ELISA	[115]

**Table 8:** Prevalence of *Mycobacterium* spp. in wild boar, feral pigs and warthog organs and tissues, 2012–2022.

Prevalence/Frequency	Species	Country	Area	Comment	Ref.
37.7% (29/77)	*M. bovis*	Brasil	Rio Grande do Sul	Sampling in 2013–2019 Test used: DNA extraction from lungs, lymph nodes, liver, spleen and kidney followed by PCR	[91]
1.1% (10/894)	*MTC*	Spain	Basque County	Sampling in 2010–2019 Test used: isolation from lymph nodes followed by real time PCR and spoligotyping of the isolates Positive cultures were detected only form head lymph nodes	[89]
2.8% (5/176)	*MTC* (mainly *M. microti)*	Switzerland	Canton of Ticino	Sampling in 2017–2018 Test used: isolation from lymph nodes + direct PCR followed by MALDI-TOF MS identification High prevalence of N-MTC identification (57.4%)	[119]
38.2% (21/55)	*M. caprae*	Poland	Bieszczady Mountains region	Sampling in 2011–2017 Test used: isolation form lymph nodes followed by PCR and spoligotyping of the isolates	[116]
76.7% (946/1235)	*Mycobacterium* spp.	Spain	Doñana National Park	Sampling in 2006–2018 Test used: Visual inspection for TBLL	[133]
1.6% (8/495) Culture 4.4% (17/386) PCR	*M. bovis*	France	Aquitaine, Côte d’Or and Corsica	Sampling 2014–2016 Test used: isolation or direct PCR form lymph nodes followed by spoligotyping of the isolates	[94]
47.1% (16/34)	*M. bovis*	South Africa	Greater Kruger National Park	Sampling in 2015 Test used: Intradermal Tuberculin Test (ITT) on captured warthog. Lymph nodes bacterial culture followed by PCR identification	[97]
2.4% (180/7634)	*M. bovis*	France	National scale (11 at-risk areas)	Sampling in 2011–2017 Test used: Lymph nodes bacterial culture followed by PCR identification Detected in 7 of the 11 at-risk areas	[98]
37.0% (25/67)	*M. bovis*	South Africa	uMhkuze Nature Reserve in Kwa-Zulu Natal, Marloth Park on the southern border of Kruger National Park in Mpumalanga	Sampling in 2013–2015 Test used: Lymph nodes bacterial culture followed by PCR identification	[96]
6.8% (19/280)	*Mycobacterium* spp.	Italy	Sicily	Sampling in 2004–2014 Test used: Visual inspection for TBLL. Tissue samples with TBLs were cultures followed by PCR identification. *M. bovis* was isolated from one sample	[100]
16.2% (647/3963)	*Mycobacterium* spp.	Portugal	Idanha-a-Nova	Sampling in 2006–2016 Test used: Visual inspection for tuberculosis-like lesions (TBLL). Considered positive when at least in one organ or lymph node showed TBLs	[129]
4.3% (329/7729)	*MTC*	Spain	Castilla y León	Sampling in 2011–2015 Test used: Lymph nodes bacterial culture followed by PCR identification	[134]
2.5% (3/118)	*M. bovis*	South Korea	Gyeonggi Province	Sampling in 2011–2015 Test used: Lymph nodes and lung bacterial culture followed by PCR identification	[102]
38.3% (16/41)	*M. bovis*	Portugal	Castelo Branco	Sampling in 2009–2013 Test used: first screening by visual inspection for TBLL (41/192 had lesions). Tissue samples with TBLs were cultures followed by PCR identification.	[105]
18.2% (8/44)	*Mycobacterium* spp.	Slovenia	Different areas	Sampling in 2010–2013 Test used: Lymph nodes and liver bacterial culture followed by PCR identification. No MTC were isolated	[130]
13.5% (36/267)	*M. caprae*	Hungary	South-Western Hungary	Sampling in 2008–2013 Test used: bacterial culture followed by PCR identification.	[117]
33.9% (18/58)	*M. bovis*	Spain	Sevilla province	Sampling in 2012–2013 Test used: Lymph nodes bacterial culture followed by PCR identification and spoligotyping. The study was performed on wild boar piglets	[108]
0% (0/9)	*M. bovis*	Brasil	Pantanal area	Test used: bacterial culture of unspecified feral pigs´ tissues followed by PCR identification	[145]
25.2% (61/242) PCR 21.5% (52/242) RPFL	*MTC*	Italy	Lombardia Region	Sampling in 2002–2003 Test used: Lymph nodes histology, bacterial culture, PCR, RFLP *M. microti* in 52 samples and *M. bovis* in 2 samples by RFLS	[123]
8.5% (51/602) PCR 5.8% (35/602) RFPL	*M. microti*	Italy	Lombardia Region	Sampling in 2006 Test used: Lymph nodes histology, bacterial culture, direct PCR, direct RFLP	[123]
7.5% (23/307) Culture 64.2% (197/307) PCR 55.0% (169/307) RFPL	*M. microti*	Italy	Lombardia Region	Sampling in 2007–2011 (only wild boar with TBLL) Test used: Lymph nodes histology, bacterial culture, direct PCR, direct RFLP	[123]
59% (1512/2562)	*Mycobacterium* spp.	Spain	Ciudad Real province	Sampling in 2008–2012 Test used: Visual inspection for TBLL in lymph nodes and organs. Generalised TBLs were detected in 51% of the subjects	[146]
2.59% (33/1275)	*MTC*	Spain	Asturias and Galicia	Sampling in 2008–2012 Test used: lymph nodes and organs culture followed by PCR identification and spoligotyping of the isolates Number of *M. bovis* isolates = 19 and *M. caprae* isolates = 14	[111]
3.64% (6/165)	*MTC*	Switzerland and Liechtenstein	Geneva, Thurgovia, Saint Gall, Grisons, Tessin, Liechtenstein	Sampling in 2009–2011 Test used: lymph nodes and tonsil culture followed by PCR identification and spoligotyping of the isolates	[124]
37.3% (293/785)	*M. bovis*	New Zealand	Different areas	Sampling in 1997–2007 Test used: Lymph nodes culture followed by PCR identification	[114]
88.9% (16/18)	*M. bovis*	Spain	Andalusia	Sampling in 2006–2010 Test used: Culture of pool homogenate of lymph nodes and lungs followed by PCR and spoligotyping of the isolates	[115]
13.3% (2/15)	*M. bovis*	Italy		Test used: Culture and PCR of swab samples on muscle surface of wild boar without TBLL	[142]
8.7 R_0_	*Mycobacterium* spp.	Spain and Portugal	29 sites	Metadata analyses from 2010–2019. Test used: gross pathology and culture Reproduction number (R_0_) defined considering prevalence in the host species, MTC excretion in infected host species, abundance of the host species, transmission rate to host species	[138]

#### 3.2.6. *Salmonella*

Salmonellosis is an enteric infection caused by species of the *Salmonella* genus other than *Salmonella* Typhi and *Salmonella* Paratyphi. Salmonellae are gram-negative bacteria belonging to the Enterobacteriaceae family. They are motile, nonsporeforming, aerobic, or facultatively anaerobic. The transmission of this infection occurs principally by the fecal-oral route: the ingestion of contaminated food or water, contact with infected animals, feces or contaminated environments. The main symptoms of salmonellosis are diarrhea, abdominal cramps, vomiting, and fever. The severity and course of the disease are related to the serotype, the number of microorganisms ingested, and the individual’s immune system [147]. *Salmonella* spp. is widely spread for its ability to infect several animal species and survive in different environmental conditions with a wide range of temperatures (2–54 °C) and pH values (3.7–9.4) [148].

Salmonellosis is a public health issue, and it was the second zoonosis reported in the EU in 2021, with 60,050 confirmed human cases, 11,785 hospitalisations, and 71 fatalities [15]. The *Salmonella* genus consists of two species: *Salmonella bongori* and *Salmonella enterica*, the latter divided into six subspecies and several serotypes [149]. The main *Salmonella* serovars implicated in human infections in 2020 and 2021 were *S.* Enteritidis, *S.* Typhimurium, monophasic *S.* Typhimurium (1,4, [5],12:i:-), *S.* Infantis, and *S.* Derby [15,150].

Overall, 80 articles regarding *Salmonella* in wild boars have been found in the literature from 2012 to 2022, seven of which are reviews [10,11,150,151,152,153,154,155], and 28 articles were not considered relevant for this study. The prevalence of *Salmonella* in the wild boar population has been studied through the analysis of different matrices. Some authors investigated the seroprevalence from blood serum, diaphragm, or muscle samples, achieving different percentages: 1.27% (141/1103) [156], 3.6% (14/393) [157], 4.3% (4/94) [158], 5% (1/20) [159], 17% (21/126) [160], 19.3% (52/269) [161], 38% (69/181) [39], and 66.5% (255/383) [162]. Testing of serum samples can reveal the presence of antibodies against *Salmonella* spp. in wild boars but not the presence of the microorganism on carcass surfaces or meat. The prevalence of *Salmonella* spp. in other matrices such as feces, spleen, kidney, submandibular lymph nodes, ileocecal lymph nodes, mesenteric lymph nodes, and tonsils is reported in Table 9, which shows that feces are the main investigated samples with a prevalence range of 0% to 43%. As shown in Table 10, the prevalence of *Salmonella* spp. in wild boar carcasses is between 0% and 2.5%, while in meat samples it ranges from 0% to 35.7%. This wide variability could be due to different geographic sampling areas, sampling methods, and the hygienic level of process procedures and the environment. The presence of *Salmonella* in wild boar cured meat products was investigated only by Roila et al. [83] in wild boar salami. *Salmonella enterica* serovar typhimurium and *Salmonella enterica* serovar Rissen were found in different batches of meat batter and salami after 7 days of curing, but in the final product after 60 days of aging, *Salmonella* spp. were not detected. However, it was not possible to specify if wild boar had been the source of *Salmonella* since the salami were made with 50% wild boar meat and 50% pork meat.

In order to reduce the risk of infection, it is recommended to pay particular attention to the skinning and evisceration processes, maintain the cool chain, have a good hygienic level during meat cutting, and to cook the final product.

#### 3.2.7. *Staphylococcus aureus*

*Staphylococcus aureus* is a gram-positive, spherical, nonsporeforming, coagulase-positive, aerobic or anaerobic, facultative, halophilic bacterium with the tendency to aggregate in “grape-like” clusters. The usual habitat of this commensal microorganism is the skin and nose of healthy humans and animals, but in some cases, it could lead to a wide range of clinical infections such as bacteremia, endocarditis, pneumonia, infections of the skin and soft tissues, mastitis, and bone and joint infections [182,183]. Some *S. aureus* strains may develop resistance to beta-lactam antibiotics, which are widely used to treat infections, and these strains are termed methicillin-resistant *Staphylococcus aureus* (MRSA). MRSA used to be associated mainly with hospital-related infections, but recently this strain has been found also in people without any contact with hospitals and, in companion animals, livestock, and wild animals [184]. There is an increasing interest in understanding the role of wild boars as possible reservoirs of *S. aureus* and MRSA in particular. About this topic, it has been found in 27 articles from 2012 to 2022, 14 of which were relevant for this study. The majority of studies performed nasal swabs for the detection of *S. aureus*, with a variable prevalence as shown in Table 11. Sousa et al. [185] considered both oral and nasal swabs, with a prevalence of *S. aureus* of 33%. Both studies from Porrero et al. [186,187] considered skin and nasal swabs; in the first study, they found 0.86% of animals positive for MRSA, of which 62.5% were detected from skin swabs and 37.5% from nasal swabs, and only one wild boar was positive in both the skin and nasal samples. Instead, Porrero et al. [187] noticed a higher percentage of positives for *S. aureus* in the nasal sample rather than in skin swabs, but without skin swabs, 18.25% of positives for wild boars would not have been detected. Only Traversa et al. [188] considered lymph nodes for the detection of *S. aureus* in wild boar and revealed a prevalence of 3.2%. No studies on the presence of *S. aureus* in carcasses, raw meat, or processed meat were retrieved in our literature survey.

#### 3.2.8. Verotoxinogenic/Shigatoxinogenic *E. coli*

Verotoxinogenic/Shigatoxinogenic *E. coli* (VTEC/STEC) form a group of pathogenic *E. coli* (gram-positive short-rods) that elaborate Shiga-like toxins together with other virulence factors. Infections in humans can range from bloody diarrhea to life threatening coagulopathy and renal failure/hemolytic-uremic syndrome. Originally associated with the presence of the O157 antigen, a number of strains with other O-serotypes have been identified as STEC. It has been proposed to use *stx*-gene typing to assess the pathogenicity of STEC (EFSA 2020). In particular, *E. coli* with genes encoding for the stx-2 gene and the virulence factor intimin (*eae*) are associated with severe courses of the disease [15]. In 2021, 6084 confirmed cases were reported in the EU, with 901 hospitalizations and 18 fatalities. From the 5 strong evidence outbreaks, 3 were attributable to meat or meat products [15]. In many animal species, asymptomatic STEC carriers are the rule. In particular, ruminants do not show symptoms since they lack vascular receptors for the Shiga-toxins [195]. A survey of notifications in the RASFF revealed no cases of wild boar meat contamination with STEC.

As regards wild boar, the literature search retrieved 27 documents. The definitions for pathogenic *E. coli* were not consistent between the studies. In 12 studies, the prevalence of STEC was reported, ranging from 0 to 28.3% (Table 12). Data on meat were reported in merely four studies, with a prevalence ranging from 0 to nearly 43% (Table 13). A more detailed view of other isolates with pathogenic potential and antimicrobial resistance described in the studies is outside the scope of our review. E.g., one study reported the isolation of STEC from wild boars with the additional feature of producing enterotoxins (*sta1* and *stb* genes), causing oedema disease [196].

Three studies reported the transmission of STEC from the feces of wild boar to fresh produce [197,198] or to recreational waters [199]. Although not the primary focus of this review, the studies highlight indirect transmission routes of pathogenic bacteria to humans.

**Table 12:** Prevalence of Shiga toxin-forming *E. coli* in wild boar, fecal samples, lymphatic organs, 2012–2022.

Prevalence/Frequency	Species	Matrix	Country	Comment	Ref.
14% (8/56)	*STEC (stx2)*	Feces	Portugal	Culture and PCR, WGS	[200]
6.9% (37/536)	*STEC*	Feces	Germany	Culture, PCR	[167]
1.9% (9/474)	*STEC* O157	Feces	Japan	Culture, PCR	[201]
6.5% (13/200)	*STEC*	Feces	Italy (Tuscany)	Culture, PCR	[202]
1.2% (3/248)	*STEC*	Feces	Japan	Culture, PCR	[56]
28.3% (43/152)	*STEC*	Feces	Poland	Culture, PCR; includes STEC and AE-STEC	[203]
4.8% (1/21)	*STEC*	Feces	Portugal	Culture, PCR	[204]
3.33% (3/90)	*STEC*	Feces	Spain	Culture, PCR	[205]
3.4% (4/117)	*E. coli* O157	Feces	Spain	Culture	[206]
0% (0/88)	*E. coli* O157:H7	Tonsils, lymph nodes, feces	Finland	Culture, PCR	[172]
0% (0/121)	*STEC* O157, O26	Feces	Japan	Culture, PCR	[67]
0% (0/301)	*STEC* O157	Feces	Spain	Culture, PCR	[57]

**Table 13:** Prevalence of Shiga toxin-forming *E. coli* in wild boar meat and carcasses.

Prevalence/Frequency	Species	Matrix	Country	Comment	Ref.
42.9% (12/28)	*STEC (stx1+ stx2+eae)*	Meat (foreleg)	Italy (Campania)	Culture, PCR (27/28 *eae* positive)	[64]
0% (0/128)	*STEC*	Meat	Japan	Culture	[179]
0% (0/310)	*STEC* O157	Meat	Spain	Culture, PCR	[57]
5.3% (3/57)	*STEC*	Meat and meat products	Spain	Culture, PCR	[207]

#### 3.2.9. Yersinia

The Enterobacteriaceae family includes the food-borne pathogen *Yersinia enterocolitica,* responsible for yersiniosis in humans, a gastrointestinal disease that could simulate appendicitis and can cause mesenteric lymphadenitis, reactive arthritis, erythema nodosum, and conjunctivitis [208,209]. The disease appears to be widespread, with ca. 6800 cases in Europe in 2020 and 100,000 illnesses every year in the USA [EFSA, 2022; CDC, 2016] [15,210]. The epidemiological situation could be even more severe, as the role of biotype 1A in human infection and disease symptoms (considered non-pathogenic compared to biotypes 1B, 2,3,4 and 5) is still debated and therefore underestimated [211].

Ready-to-eat foods are the major sources of human infection, especially as *Y. enterocolitica* can resist cold environments and even replicate at refrigeration temperatures [211]. Animals, especially pigs, are considered the main reservoir of the bacteria, which could be found mainly in the intestine and tonsils [212]. Nevertheless, the outbreaks reported in 2021 are related to prepared dishes and ready-to-eat vegetables [15], and no reports are available on wild boar meat as an outbreak source.

The database research retrieved 39 studies regarding *Y. enterocolitica* in wild boars and feral pigs between 2012–2022. The articles that reported studies on the prevalence of the microorganism in animal tissue, feces, or carcasses/muscles of wild boars were 21. Only two articles describe the prevalence of antibodies against *Y. enterocolitica* in animal blood samples. Papers on *Yersinia pseudotuberculosis* were not considered. Most of the studies were conducted in Europe (19 out of 21), especially in Italy (10 articles). Samples of different matrices were considered: eight studies on fecal samples, nine on organs different from muscles, four on carcass surfaces (external or internal), and four in muscles (Table 14).

The seroprevalence in wild boar was above 50% (in Finland and the Czech Republic), proving that the microorganism is widespread in this species. Fecal material is considered the main source of contamination of the carcass and, ultimately, of the meat. This contamination could happen during hunting (the precision of the shot), evisceration, or carcass processing and cutting [176,180]. Fecal sample positivity for *Y. enterocolitica* ranges from 0% (different Italian regions) to 74% (Japan). Thus, as for other genus belonging to the Enterobacteriaceae family, the fecal shedding could be intermittent [213]. Regarding organs and tissues that could harbour the microorganism in *Suidae*, the prevalence of the microorganism in the tonsils of wild boar ranges from 14% (Sweden) to 64% (Campania Region, Italy), with a higher percentage than in lymph nodes (ranging from 0% to 4.4%). The presence of the pathogen in such tissues could be considered during carcass processing to avoid the spread of the microorganism to the meat. Nonetheless, in wild boar, in contrast to the domestic pig, the head is removed during carcass dressing at cervical vertebrae level, thus the laryngeal and pharyngeal area is removed from the carcass at an early stage of the processing chain.

The presence of *Y. enterocolitica* on carcass surfaces ranges from 0% to 85.7%. Such a wide range could be due to different sampling methods and areas, but also to differences in the hygienic level of the process. The same might hold true for muscles, where the prevalence ranges from 0% to 71%. The wide range of prevalence denotes that, although wild boar can harbour microorganisms in the intestines and tonsils, the procedures to obtain the meat are relevant to prevent contamination of muscles. In this perspective, the training of the personnel, the presence of suitable structure and equipment, the correct hygienic procedure implementation, and standard sanitation operating procedures are of paramount importance.

Another important aspect that emerged from the literature survey is that the biotype most frequently observed in wild boar is 1A, the least pathogenic but also the most underrated of the *Y. enterocolitica* biotypes.

**Table 14:** Prevalence of *Yersinia enterocolitica* in wild boar, feral pigs and warthog.

Prevalence/Frequency	Country	Area	Matrix	Comment	Ref.
0% (0/107)	Italy	Valle d’Aosta Region	Feces	Sampling in 2015–2018 Test used: PCR	[214]
85.7% (12/36)	Italy	Campania Region	Carcass	Sampling in 2019 Test used: bacterial isolation and SYBR green PCR-assay for ystA and ystB genes. 12 animals carried ystB gene, and 3 animals both ystA and ystB genes	[62]
64.3% (9/36)			Tonsils		
71.4% (10//36)			Muscle		
0.01% (1/110)	Tunisia	Ariana, Bizerte, Manouba, Nabeul and Siliana	Feces	Sampling in 2018–2020 Test used: bacterial isolation and biochemical identification	[215]
0% (0/64)	Italy	Parma and Bologna province	Carcass and Mesenteric lymph nodes	Sampling in 2020 Test used: bacterial isolation and biochemical identification	[165]
2.6% (126/4890)	Italy	Liguria Region	Liver	Sampling in 2013–2018 Test used: bacterial isolation, Serotyping and Real Time PCR for virulence genes. Biotype 1A was the most isolated (92.9%), then biotype 1B (6.3%) and 2 (0.8%)	[216]
18.8% (54/287)	Italy	Tuscany Region	Rectal swab	Sampling in 2018–2020 Test used: bacterial isolation, biochemical identification. and Real Time PCR for virulence Genes. Identification of gene *ystA* in 14 out of 54 isolates, *inv* in 13, *ail* in 12, *ystB* in 10 and *virF* in 8	[85]
56.4% (102/181)	Finland	12 out of 19 regions	Blood	Sampling in 2016 Test used: seroprevalence ELISA test.	[39]
16.9% (22/130)			Spleen and kidneys	Test used: Organs: real-time PCR based on SYBRGreen for *ail* gene	
6.2% (19/305)	Italy	Parma and Piacenza provinces	Feces	Sampling in 2017–2019 Test used: bacterial isolation, biochemical identification, and Real Time PCR for virulence Genes. All isolates belonged to biotype 1A	[217]
3.3% (10/305)			Mesenteric lymph nodes		
74.1% (40/54)	Japan	Not specified	Feces	Sampling in 2014–2016 Test used: bacterial isolation, biochemical identification. Prevalence is reported for *Yersinia* spp. 97.4% of the *Y. enterocolitica* isolates belonged to biotype 1A	[218]
13.6% (3/22)	Italy	Campania region	Muscle	Sampling in 2017 Test used: bacterial isolation, biochemical identification, and Real Time PCR for virulence Genes. All isolates present only *ystB* gene	[178]
6.7% (6/90)	Sweden	13 counties in southern Sweden	Feces	Sampling in 2014–2016 Test used: bacterial isolation, and Real Time PCR for ail gene	[219]
14.0% (19/136)			Tonsils		
4.4% (4/90)			Mesenteric lymph nodes		
25.3% (110/434)	Poland	12 out of 16 Polish regions	Rectal swab	Sampling in 2013–2014 Test used: bacterial isolation, and multiplex PCR for ail, ystA and ystB genes. 92.5% of the isolates belong to biotype 1A	[220]
0% (0/42)	Italy	Tuscany Region	Muscle	Sampling in 2013–2014 Test used: bacterial isolation, and biochemical identification	[181]
65.9% (89/135)	Czech Republic	Moravian Regions	Blood	Sampling in 2013–2014 Test used: ELISA	[221]
55.5% (11/20)	Poland	North-East Poland	Swab samples from tonsils area, peritoneum and perineum	Sampling in 2013 Test used: bacterial isolation, and biochemical identification biotyping, serotyping and molecular characterisation. 90.5% of the isolates belong to biotype 1A	[222]
33.3% (24/72)	Spain	Basque Country	Tonsils	Sampling in 2001–2012 Test used: bacterial isolation, biochemical identification, and molecular characterization	[223]
15.3% (17/111)	Germany	Lower saxony	Tonsils	Sampling in 2013–2014 Test used: bacterial isolation, MALDI-TOF identification, Real Time PCR for virulence Genes. 89.55% of the isolates belong to biotype 1A	[224]
20.5% (18/88)	Sweden	Central Sweden	Feces and Ileocecal lymph nodes and tonsils	Sampling in 2010–2011 Test used: bacterial isolation, and multiplex PCR for *ail* gene	[219]
27.3% (18/66)	Spain	Basque Country	Tonsils	Sampling in 2010–2012 Test used: bacterial isolation, and biochemical identification and direct real time PCR with new enrichment protocol	[225]
0% (0/3)	Argentina	San Luis city	Tonsils and tongue	Sampling in 2008–2012 Test used: bacterial isolation and biochemical identification	[173]
14.8% (34/230)	Italy	Viterbo Province	Muscle	Sampling in 2012–2013 Test used: bacterial isolation, and multiplex PCR for *ail* gene	[157]
4.2% (3/72)	Italy	Upper Susa valley Piedmont Region	Carcass	Sampling in Test used: bacterial isolation, biochemical identification and molecular characterisation for *inv, ail* and *yst* genes. *ail* and *yst* genes were not detected	[86]

## 4. Conclusions

The increasing popularity of meat from wild game is observed in many countries. Diseases in wildlife have often been seen as an issue or spill-over or spill-back of infection agents from farm animals, and exposure of humans and animals in frequent and close contact with wild animals has been studied to some extent. Additionally, while the presence of antibodies against a specific pathogen may be useful for epidemiological purposes, its value for the assessment of meat safety is primarily that the given pathogen must be considered a potential hazard. Similarly, the presence of pathogens in the feces and even in the lymph nodes of the digestive tract mainly indicates that the host organism can keep the pathogen under control. Similar to farm animals, it can be expected that stress, but also the dressing procedures after killing, can cause the spread of the pathogen on/in edible organs. Since these scenarios do not result in any typical lesion, the routine ante- and post-mortem examinations [226] will not give an indication of the presence of a certain pathogen, and minimizing the spread of the agent is a matter of good hygienic practice. However, if serological or other testing has demonstrated the presence of a certain pathogen in wildlife in a certain region, it would be wise to adopt hygienic precautions (i.e., no admittance of carcasses with “gut shots” in the food chain; or disinfecting knives after cutting in the tonsillar area).

For five (*Campylobacter*, *Listeria monocytogenes*, *Salmonella*, Shiga toxin-forming *E. coli,* and *Yersinia enterocolitica*) of the nine agents we reviewed, one or more studies dealt with the presence of the pathogen on muscle surfaces or muscle tissues of wild boar, with prevalences ranging from 0 to ca. 70%. One experimental study was retrieved on the transmission and survival of *Mycobacterium* on wild boar meat. As regards edible inner organs, the liver and spleen have been examined for the presence of *Brucella*, *Coxiella burnetii*, *Listeria monocytogenes,* and *Mycobacteria*, and the latter four agents have actually been recovered, albeit with varying percentages. For *Brucella,* human case reports and epidemiological studies in (hunting) dogs stressed the occupational exposure risk, but no indication of meat-borne transmission to humans was evidenced. Similarly, the mode of transmission of *C. burnetii* is more likely via vectors (i.e., ticks). In most studies, animals without specific histories or pathologies had been examined.

In essence, the literature we reviewed confirmed that food-borne pathogenic bacteria present in meat from domestic animals [15] and implicated in food-borne disease can also be found in wild boars, with varying prevalence and regional differences. It is unclear to what extent such differences are biased by sampling and analytical procedures. In the absence of more detailed data for the European Union, it might be advisable to focus on the efficacy of current game meat inspection [226] and handling practices [140] to minimize introduction in the game meat chain. Similarly, the implementation of HACCP-based food safety management systems [227] needs to be stressed.

With respect to the placing on the market of meat from wild hunted game, European Union legislation distinguishes an “approved” chain (i.e., the hunted game specimens are collected, post-mortem inspected, and processed in approved establishments) from an unapproved chain, which is largely subject to national regulation (for primary products, i.e., the eviscerated carcass, see Recital 10 and Article 1 of EC Regulation 852/2004 [228]; for processed or unprocessed products, see Recital 11 and Article 1 of EC Regulation 853/2004 [140]). This unapproved chain represents the supply of small quantities of wild game or wild game meat directly from the hunter to the final consumer or to local retail establishments directly supplying the final consumer [140].

Currently, there is no uniform way in which this unapproved sector is regulated in the member states; there is even no consistent definition of “small quantities of wild game or wild game meat” [140]. Admittedly, all national legislation has a common baseline represented by EC Regulation 178/2002 (in particular, Articles 14, 16–19; “safe food”, traceability, identification of hazards, and management of risks) [229,230]. An in-depth and comprehensive consideration of said regulation should, in fact, be sufficient to warrant food safety. European Union member states have chosen different approaches [231,232], but there are no real metrics to assess how the systems actually perform in managing the consumers´ risk posed by the presence of foodborne pathogens in game meat.

## Figures and Tables

**Table 1:** Agents or diseases of wild boar covered in the literature survey (2012–2022), their coverage in legislation, and the number of pertinent publications.

Agent/Disease	Type	Zoonotic	EU Zoonoses Directive	OIE Listed	n, Period 2012–2022	n, period 2012–2016	n, period 2017–2022	Average /Year, Period 2012–2016	Average/Year, Period 2017–2022	Ratio of Averages
African Swine Fever	V	n		y	499	58	441	11.6	73.5	6.3
Aujeszky’s Disease	V	n		y	108	43	65	8.6	10.8	1.3
CSF	V	n		y	158	54	104	10.8	17.3	1.6
Foot and Mouth Disease	V	n		y	35	13	22	2.6	3.7	1.4
Porcine Respiratory and Reproductive Syndrome	V	n		y	62	27	35	5.4	5.8	1.1
West Nile Fever	V	n		y	17	4	13	0.8	2.2	2.7
Hepatitis A	V	y	f		0	0	0			
Influenza	V	y	f		0	0	0			
Japan Encephalitis	V	y		y	21	6	15	1.2	2.5	2.1
Rabies	V	y	f	y	19	6	13	1.2	2.2	1.8
Paratuberculosis	B	n		y	9	7	2	1.4	0.3	0.2
*Bacillus anthracis*	B	y		y	3	2	1	0.4	0.2	0.4
Borrelia	B	y	f		30	9	21	1.8	3.5	1.9
*Brucella*	B	y	m	y	95	36	59	7.2	9.8	1.4
*Campylobacter*	B	y	m		22	7	15	1.4	2.5	1.8
*Clostridium*	B	y	f (*C. botulinum*)		0	0	0			
*Francisella*	B	y		y	12	6	6	1.2	1.0	0.8
*Leptospira*	B	y	f		55	17	36	3.4	6.0	1.8
*Listeria*	B	y	m		12	3	9	0.6	1.5	2.5
Q-Fever	B	y		y	23	7	16	1.4	2.7	1.9
*Salmonella*	B	y	m		80	25	55	5.0	9.2	1.8
*St. aureus*	B	y	*		27	10	17	2.0	2.8	1.4
Tuberculosis	B	y	m (*M. bovis*), f (others)		214	97	117	19.4	19.5	1.0
Verotoxinogenic *E. coli*	B	y	m		27	10	17	2.0	2.8	1.4
*Yersinia*	B	y	f		40	13	27	2.6	4.5	1.7
*Cryptosporidium*	P	y	f		18	5	13	1.0	2.2	2.2
*Cysticercus*	P	y	f	y	9	2	7	0.4	1.2	2.9
*Echinococcus*	P	y	m	y	47	12	35	2.4	5.8	2.4
*Toxoplasma*	P	y	f		90	35	55	7.0	9.2	1.3
*Trichinella*	P	y	m	y	167	67	100	13.4	16.7	1.2

V = virus; B = bacterium; P = parasite; f = facultative, according to the epidemiological situation; m = mandatory; * = multi-resistant *St. aureus*.

**Table 5:** Presence of *Coxiella burnetii* or antibodies in wild boar or in vectors associated with wild boar, according to country and continent, 2012–2022.

Prevalence/Frequency	Matrix	Country	Comment	Ref.
0% (0/100)	Spleen	Italy (Central)	PCR	[73]
0% (0/93) 0% (0/176)	Spleen Ticks	Italy	PCR	[74]
5.1% (6/117)	Blood of dogs	Italy (Central)	PCR	[72]
0.48% (2/411)	Ticks	Italy (South)	Ticks collected from hunters and dogs	[71]
0% (0/40) feeding ticks 0% (0/489) questing ticks	Ticks	Spain (Northwest)	PCR	[75]
5.5% (4/73)	Serum	Spain (Northwest)	antibodies	[70]
1.9% (9/484)	Spleen	Spain (North)	PCR	[69]
0% (0/2256) 0% (0/167)	Ticks Spleen	Spain	Near to Barcelona, a highly populated area	[76]
0% (0/8)	Serum	Kenya	antibodies Serology (ELISA)	[41]
0% (0/67)	Blood	Brazil		[77]
5% (4/79)	Ticks	Thailand	PCR	[78]
18.3% (19/104)	Serum of dogs	Australia	Queensland	[79]

**Table 9:** Prevalence of *Salmonella* spp. in wild boar, feces, lymphatic tissues, and inner organs, 2012–2022.

Prevalence/Frequency	Species	Matrix	Country	Comment	Ref.
3.1% (13/425) 0.2% (1/425)	*Salmonella* spp. *Salmonella* spp.	Feces Mesenteric lymph nodes	Serbia	*S.* Enteritidis was the main serotype identified	[163]
3.1% (4/130)	*S. enterica*	Feces	Spain	Serotype identified were monophasic *S*. Typhimurium, *S.* Bardo, *S.* Enteritidis	[59]
35.6% (32/90) 17.8% (16/90)	*Salmonella* spp. *Salmonella* spp.	Feces Lymph nodes	Italy	46.7% (42/90) animals were positive in feces or lymph nodes, of which 11.9% (5/42) were positive at the same time in both matrices. *S.* Abony, S. Newport, *S.* Agona, *S.* Derby, *S.* Hermannswerder, *S.* Saintpaul, *S.* Elomrane, *S*. *salamae* were identified	[164]
7.8% (5/64) 4.7% (3/64)	*Salmonella* spp. *Salmonella* spp.	Mesenteric lymph nodes Carcass	Italy	Sampling from game-handling establishment, game collection point and slaughterhouse	[165]
6% (260/4335)	*Salmonella* spp.	Liver	Italy	Sampling in 2013–2017. Isolated strains belonged to all six *Salmonella enterica* subspecies and the main serotype was *S.* Enteritidis	[166]
4.18% (12/287)	*Salmonella* spp.	Liver or spleen or rectal swab	Italy	*S. diarizonae*, *S. houtenae*, *S.* Newport, *S.* Kottbus, *S.* London, *S.* Infantis, *S.* Rubislaw were identified.	[85]
2.4% (13/552)	*Salmonella* spp.	Feces	Germany	*S.* Enteritidis, *S.* Typhimurium, *S.* Stanleyville, were identified	[167]
5% (6/130)	*Salmonella* spp.	Spleen and kidney	Finland		[39]
0% (0/115)	*Salmonella* spp.	Feces	Denmark		[52]
15.9% (30/189) 3.2% (6/189)	*Salmonella* spp. *Salmonella* spp.	Mesenteric lymph nodes Feces	Italy	Three animals were positive in both samples	[168]
18.69% (40/214) 5.06% (21/415) 2.98% (25/838)	*Salmonella* spp. *Salmonella* spp. *Salmonella* spp.	Tonsils Submandibular lymph nodes Feces	Spain	Sampling in 2010–2015 From 148 wild boars the 3 matrices were collected in the same animals and 27.02% (40/148) of them were positive to *Salmonella* spp. (31/148 tonsils, 12/148 lymph nodes, 2/148 feces) but none of them were positive in the three samples simultaneously	[169]
7% (4/57) 3.5% (2/57)	*S. enterica* *S. enterica*	Feces Mesenteric lymph glands	Italy	*S.* Thompson and *S.* Braenderup were identified	[63]
43.9% (194/442)	*Salmonella* spp.	Feces	USA	Sampling from 2013 to 2015. Main serovars identified were *S. Montevideo*, *S. Newport* and *S. Give*	[170]
5% (1/21)	*Salmonella* spp.	Feces	Portugal		[171]
5.1% (9/175) 1.8% (1/56) 1.1% (1/88)	*Salmonella* spp. *Salmonella* spp. *Salmonella* spp.	Tonsils Ileocaecal lymph nodes Feces	Sweden	*S. enterica* and *S. diarizonae* were identified	[172]
33.3% (1/3) 33.3% (1/3)	*Salmonella* spp. *Salmonella* spp.	Tonsils Tongue	Argentina	Tonsils carried both *S.* Gaminara and *S.* Newport, while only *S.* Gaminara were isolated from tongue	[173]
5% 2/40	*S. enterica*	Feces	Spain	*Salmonella enterica* serotype Anatum and Corvallis were isolated	[61]
7.4% (9/121)	*Salmonella* spp.	Feces	Japan	*S. enterica* subsp. *enterica* serovar Agona (3), *S.* Narashino (2), *S.* Enteritidis (1), *S.* Havana (1), *S.* Infantis (1), and *S.* Thompson (1) were obtained	[67]
0.3% (1/333)	*Salmonella* spp.	Feces	Spain	One animal was positive in both carcass and feces samples. *S.* Bardo, S. Montevideo, *S. arizonae III* (16:i,v:1,5,7) and *S.* Typhimurium were identified	[57]
10.8% (54/499)	*Salmonella* spp.	Feces	Italy	*S. enterica* subsp. *salamae II*, *S. enterica* subsp. *diarizonae III b*, *S. enterica* subsp. *houtenae IV* and *S*. Fischerhuette were the most common isolated	[162]
24.82% (326/1313)	*Salmonella* spp.	Feces	Italy	Sampling from 2007 to 2010 *S. enterica* subsp. *enterica* was the main serovar isolated (79.5%)	[174]
15.4% (33/214)	*Salmonella* spp.	Feces	Spain		[175]

**Table 10:** Prevalence of *Salmonella* spp. in wild boar meat and carcasses, 2012–2022.

Prevalence/Frequency	Species	Matrix	Country	Comment	Ref.
2.7% (1/36) 0% (0/36)	*Salmonella* spp. *Salmonella* spp.	Meat Carcass	Italy		[62]
35.7% (10/28)	*Salmonella* spp.	Meat	Italy	*S.* Veneziana, *S.* Kasenyi, *S.* Coeln, *S.* Manhattan, *S.* Thompson and *S.* Stanleyville were identified	[64]
2.5% (3/121)	*Salmonella* spp.	Carcass	Italy	Two *S.* Stanleyville and one *S.* Typhimurium were identified	[176]
1.1% (1/90)	*Salmonella* spp.	Carcass	Italy		[164]
0% (0/37)	*Salmonella* spp.	Meat	Italy	Meat cut sampled were fillet and legquarter	[177]
31.82% (7/22)	*Salmonella* spp.	Meat	Italy	*S.* Stanleyville, *monophasic S.* Typhimurium, and *S.* Kasenyi were identified	[178]
0% (0/30)	*S. enterica*	Carcass	Italy		[63]
0% (0/128)	*Salmonella* spp.	Meat	Japan		[179]
1.4% (3/210) 1.9% (4/210)	*Salmonella* spp. *Salmonella* spp.	Skin Carcass	Serbia		[180]
4.55% (1/22)	*Salmonella* spp.	Meat	Italy	Meat cut sampled was *Longissimus dorsi* muscle	[181]
1.2% (4/333)	*Salmonella* spp.	Carcass	Spain	One animal was positive in both carcass and feces samples	[57]
0% (0/72)	*Salmonella* spp.	Carcass	Italy		[86]

**Table 11:** Prevalence of MRSA on wild boar mucosal membranes and in lymphatic organs, 2012–2022.

Prevalence/Frequency	Species	Matrix	Country	Comment	Ref.
36.9% (41/111)	*S. aureus*	Nasal swab	Germany	MRSA were not detected	[189]
33% (30/90)	*S. aureus*	Oral and nasal swab	Portugal	7 isolates showed resistance to at least one of the antibiotics tested; 1 MRSA CC398 (spa-type t899) was identified	[185]
32.2% (57/177)	*S. aureus*	Nasal swab	Portugal	Isolates were resistant to all antimicrobials tested, except of trimethoprim-sulfamethoxazole and vancomycin	[190]
17.8% (66/371) 13.7% (51/371) 1.96% (1/51)	CoPS *S. aureus* MRSA	Nasal swab	Spain	74.5% isolates were susceptible to all the antimicrobials analyzed, 19.6% were resistant to penicillin and 9.8% were resistant to streptomycin	[191]
17.67% (126/713)	MSSA	Skin and/or nasal swabs	Spain		[187]
6.8% (8/117)	*S. aureus*	Nasal swabs	Germany	No antibiotic resistance was detected	[192]
3.2% (23/697)	*S. aureus*	Lymph nodes	Italy	MRSA were not detected	[188]
0.87% (5/577)	MRSA	Nasal swab	Germany		[167]
0.86% (7/817)	MRSA	Skin and nasal swabs	Spain	8 isolates were identified from 7 positive animals: 3 from nasal swabs and 5 from skin swabs. One animal was MRSA positive for both skin and nasal swabs	[186]
0% (0/90)	MRSA	Nasal swab	Spain		[193]
0% (0/439)	MRSA	Nasal swab	Germany		[194]
0% (0/244)	MRSA	Nasal swab	Denmark		[52]

MRSA: methicillin-resistant *Staphylococcus aureus*; MSSA: methicillin-susceptible *Staphylococcus aureus* (MSSA); CoPS: coagulase positive *Staphylococcus.*

## Data Availability

Not applicable.
